# Sunflower Meal Inclusion Rate and the Effect of Exogenous Enzymes on Growth Performance of Broiler Chickens

**DOI:** 10.3390/ani12030253

**Published:** 2022-01-21

**Authors:** Mbuso Jethro Mbukwane, Thobela T. Nkukwana, Peter W. Plumstead, Natasha Snyman

**Affiliations:** 1 Department Wildlife and Animal Sciences, University of Pretoria, Pretoria 0002, South Africa; thobela.nkukwana@up.ac.za; 2 Chemuniqué (Pty) Ltd., 28 Eagle Lane, Lanseria Business Park, Lanseria 1739, South Africa; peter@chemunique.co.za (P.W.P.); Natasha.S@chemunique.co.za (N.S.)

**Keywords:** cage effect, enzyme supplementation, non-starch polysaccharides, production performance, Ross 308

## Abstract

**Simple Summary:**

Over the years, there has been an increase in the price of traditionally used protein sources such as soybean meal (SBM) in broiler feed. This has necessitated the need for alternative protein sources that can partially substitute the SBM protein and reduce the cost of feeding. Sunflower meal (SFM), a by-product from the oil processing industry, is available in significantly high quantities throughout the year at lower cost. SFM can be produced in drought-stricken places with good harvest and is less prone to fungal infestation. Although SFM has protein levels ranging between 32 and 37 percent, its inclusion levels in broiler diets have been limited to 5% due to high concentration of non-starch polysaccharides, low metabolizable energy and lysine levels. This presents challenges in feed manufacturing, since composition depends on the amount of oil extracted and retained hulls, with effects on the digestive efficiency of broiler chicks, especially in the first 21 d of growth. The use of multi-enzymes that target specific substrates in SFM can potentially allow an increase in its inclusion levels, thereby reducing the deleterious anti-nutritional effects from non-starch polysaccharides (NSPs). Additionally, multienzyme supplementation can result in flock uniformity and environmental pollution reduction due to less nutrient loss in manure.

**Abstract:**

The study examined the effect of de-hulled sunflower meal (SFM) inclusion rate and exogenous enzymes (EE) on broilers production performance. A four-feeding phase of pre-starter (1–9 d), grower (10–20 d), finisher (21–28 d) and post-finisher (29–35 d) was used with SFM included as low (BSL) and high (BSH) in all phases. BLS inclusion was 3% throughout phases and BSH inclusion was 7.5%, 10%, 13% and 13.5% for the 4-phases. Each SFM had a negative control (NC) (BSL− and BSH−) and positive (PC) (BSL+ and BSH+) control with additional 80 kcal Apparent Metabolizable Energy. Enzymes: xylanase (X), xylanase + beta-glucanase (XB), xylanase + beta-glucanase + protease (XBP) and xylanase + amylase + protease (XAP) were added to the NC and PC to give 6 treatments. Pen body weight gain (BWG) and feed intake (FI) were determined at 9, 20, 28 and 35 d and feed conversion ratio (FCR) was calculated accordingly. Diets were fed ad libitum to 1920 male Ross 308 broilers. Diet type, enzyme and diet by enzyme interactions were not significantly different amongst treatment diets. During the pre-starter and the grower phase, all studied parameters did not significantly differ from each other. All studied parameters were significantly influenced by enzyme addition and diet-type and enzyme interaction at 35 d except for diet type on FCR. Broilers fed BSH supplemented with XAP recorded the highest BWG (2.69 kg), whereas broiler chickens on BSL and supplemented with XBP recorded the lowest BWG (2.60 kg). SFM can be increased to 13% and 13.5% finisher and post-finisher diets without negatively affecting performance, and X and XAP enzymes can improve BWG of broilers grown to 35 d.

## 1. Introduction

In modern poultry production, feed costs cover about 70–75% of total costs of production, with maize and soybean meal being the mostly used conventional feed ingredients [1,2]. Future predictions on use of soybean meal use as a protein source for both humans and animals pointed out potential problems mainly due to factors such as availability, the risk of over-reliance on a single ingredient and production costs [3]. The use of non-conventional feed materials could sustain the poultry industry by alleviating the shortage of feed materials [4]. The need for such conventional feed materials have never been so real than the present era that humankind world-over finds themselves in. Since the outbreak of the coronavirus disease (COVID-19) pandemic, we have experienced a 2% decrease in production; as well as global chicken meat reduction of 1% [5]. Among other reasons, these reductions may be due fluctuations of poultry supplies and feeds. Consequently, nutritionists are constantly in search of alternative feed ingredients that are readily available, affordable and nutritious [1]. Sunflower meal (SFM), an inexpensive by-product of agro-industry origin is one promising alternative feed ingredient that can partially replace the inclusion of soybean meal in poultry diets [6]. It is of broad availability globally, due to its wide adaptability to a range of soil and climatic conditions, it is rich in crude protein content, methionine, and have limited antinutritional factors [7,8,9]. However, its use in broiler feed has been limited by low levels of lysine and high crude fibre, high non-starch polysaccharides concentrations, low metabolizable energy content and some presence of chlorogenic acid [10,11,12,13].

Some studies indicated an inclusion level of 15% SFM in broiler diets without any negative effects on broiler performance and/or other measured parameters [14,15,16], whereas some studies reported that it can be used at higher levels with no adverse effects on utilization and growth performance of broiler chickens [11,17,18], especially with the addition of enzymes [6].

On the contrary, there were studies which reported that the use of 10% and more SFM in broiler diets negatively affected the growth performance [19,20,21,22]. Additionally, Ref. [23] reported that SFM can be used up to 20% in growing quail diets without negatively affecting performance. In their study [24], postulated that the inclusion of SFM at higher levels may necessitate the need of supplementation with synthetic lysine and oils in the diet in-order to compensate for the low metabolizable energy (ME) associated with this ingredient. However, supplementation with extra fats to rectify the low ME associated with SFM use must be done with caution, and huge attention in terms of fats storage must be exercised due to rancidity and poor pelleting quality; which may in turn necessitate the need for additional supplementation with antioxidants [14,24].

According to [11], supplementation of exogenous enzymes in poultry diets with SFM can decrease their deleterious effects and stimulate fibre digestion. Amongst other benefits of enzyme supplementation, Ref. [25] reported that enzymes functions in the breakdown of NSP’s, reduction of gut viscosity thereby improving nutrients digestibility and gut performance.

Exogenous enzymes have been used with mixed results as reported in previous studies. In some studies, growth performance parameters were worsened as a result of enzyme supplementation [8,26]; in others, it led to no improvements [6,27,28,29], whereas in others, it led to an improvement in growth performance parameters [22,23,24,26,30]. These contrasting findings created a knowledge gap and inconsistency in the industry regarding the optimum inclusion rates of SFM in poultry diets and the best enzyme combinations for optimum results.

To our knowledge, there is still a knowledge gap that exists in the optimum inclusion levels of sunflower meal in broiler diets supplemented with exogenous enzymes. Hence, the present study was conducted to determine if inclusion levels of de-hulled sunflower meal can be increased, and if the efficacy of exogenous enzymes (EE) on broiler performance is altered by sunflower meal inclusion rates.

## 2. Materials and Methods

### 2.1. Ethical Approval

The use of animals was approved by the Animal Ethics Committee at the University of Pretoria (Project No. EC042-18).

### 2.2. Experimental Diets and Treatments

A total of one thousand nine hundred and twenty (1920) 1-day old male Ross 308 broiler chicks were purchased from a local commercial hatchery to be used in the study evaluating the response of broilers to different levels of de-hulled SFM meal inclusion supplemented with exogenous enzymes. The trial was conducted in two environmentally controlled broiler houses located at the University of Pretoria’s Experimental farm, with 96 pens, 48 pens per house. The houses are equipped with both heating and cooling facilities and these becomes vital when temperatures peaks or drops and there is a control room equipped with an Orskov computer system to monitor the fluctuations in house temperatures. House environment was controlled by a combination of electric heaters, automated electric exhaust and stirring fans with mist sprayers. Minimum ventilation was always maintained ensure clean air inside the house and also to prevent accumulation of gases such as ammonia. Additionally, temperatures were daily taken twice (morning and afternoon) at similar times and on three different spots in the pens using a digital infra-red gun thermometer. On arrival, day-old broiler chicks, weighing (42.4 ± 0.23 g/chick) were placed in concrete floor pens that were bedded with fresh wood shavings to the depth of 10 cm, measuring 2 m by 1 m and 20 chicks individually lodged in each pen. Placement of birds followed a randomized block design. This was for both houses, meaning that each house had 960 birds placed randomly on the 48 pens. Each house had treatment diets randomly assigned to pens, replicated 4 times to give the 8 replicates per treatment. The vaccinations program for Marek’s disease and infectious bursal disease was followed at the hatchery prior to collection, and no vaccines were administered during the rearing period.

Birds were housed in the floor pens until the end of the experiment at day 35. Brooding temperatures for both houses was initially controlled at 34 °C for the first 4 d prior to placement, and then gradually lowered by 3 °C per week to reach approximately 22 °C and was maintained at this level until the end of the experiment. Light was continuously on during the first day. The next day a schedule of 22 h light and 2 h dark was used. During the remaining experimental period, a schedule of 14 h light, 4 h dark, 4 h light, 2 h dark was used. During the first week of the trial, chicks were fed from a combination of pen and tube feeders, and both nipple and fountain drinkers supplied water to the chicks. Feed was provided in crumble form during the starter phase (1–9 d), whereas during the remaining phases, it was supplied in pelleted form of 3 mm pellets. Daily inspection of chicks was done by removing of dead birds if any and recording of mortalities (pen number, date and body weight). Throughout the experimental period, feed and cool fresh water was provided ad libitum to the birds and feeders were always kept full to ensure that feed intake was not affected by low levels of feed.

The experiment adopted a four-feeding phase regime of pre-starter (1–9 d), grower (10–20 d), finisher (21–28 d) and post-finisher (29–35 d) phase. The sunflower meal used in the study was de-hulled sunflower meal with a crude protein (CP) of 38%. The basal diets contained low sunflower meal (BSL) and high sunflower meal (BSH). Each SFM level had a negative control (NC) (BSL− and BSH−) and positive (PC) (BSL+ and BSH+) control with additional 80 kcal Apparent Metabolizable Energy (AMEn) and no enzyme was added to this. The BSL treatment contained 3% SFM throughout the four-feeding phase adopted in the experiment. The BSH contained 7.5%, 10%, 13% and 13.5% SFM for the pre-starter, grower, finisher and post-finisher phase. Additionally, commercial enzymes; xylanase (X), xylanase + beta-glucanase (XB), xylanase + beta-glucanase + protease (XBP) and xylanase + amylase + protease (XAP) were added to the NC and PC formulated diets to give 6 dietary treatments at each SFM inclusion level. Each treatment was replicated 8 times. Experimental diets (Table 1 and Table 2) were supplied from day 1 until day 35. Phytase enzyme, (contains 1000 U phytase per kg according to manufacturer) was added at 150 g/tonne across all treatment diets as a standard industry procedure. The enzyme XB was included at 111 g/tonne, whereas, X, XAP and XBP was added at 50, 100 50 g/tonne respectively. According to the manufacturer, XB enzyme contains 1-220 U xylanase and 152 U β-glucanase per kg. XAP enzyme contains 2000 U xylanase, 200 alpha-amylase and 4000 protease per kg. The enzymes were added on top of the diets. All diets met or exceeded nutrient requirements of broilers according to [31].

Using the methods of the Association of Official Analytical Chemists [32], a proximate analysis was done (Table 3 and Table 4) on all the experimental diets and for crude protein (CP), dry matter (DM) ash, ether extract (EE) and crude fibre (CF) composition. Prior to analysis, the experimental diets were milled to pass through a 0.5 mm sieve. Dry matter was determined by drying the samples in replicates until they reached a constant weight in a forced draft oven set at 105 °C [32], method 934.01. The Dumas combustion procedure (Leco FP-428; Leco Corporation, St Joseph, MI, USA) was followed for the determination of nitrogen (N) content with EDTA as a calibration standard ([32], method 968.06). CP was calculated as N X 6.25. Crude fibre was determined using the Fibre-Tech apparatus [33].

### 2.3. Growth Performance

Pen bird body weight gain (BWG) and feed intake (FI) were monitored and recorded at 9, 20, 28 and 35 d. These were used to compute the feed conversion ratio (FCR) per phase by dividing the pen feed intake by pen body weight gain over the duration of that feeding phase.

### 2.4. Statistical Analysis

Data were analyzed statistically as a randomized block design with the Proc mixed model [34] using pen as an experimental unit for the average effects over time. Repeated Measures Analysis of Variance with the Proc mixed model was used for repeated period measures. Means and standard errors were calculated, and significance of difference (*p* < 0.05) was determined by Tukey’s test.

The Proc mixed model used is described by the following equation:

*Yijkl* = *μ* + *Ti* + *Lj* + *TLij* + *Bk* + *Hl* + *Eijkl*

where *Y* = variable studied during the period (growth parameters, i.e., BWG, FI, FCR)

*μ* = overall mean of the population
*T* = effect of the ith treatment
*L* = effect of the jth level
*TL* = effect of the ijth interaction between treatment and level
*B* = effect of the kth block
*H =* effect of the lth house
*E* = random error with each Y

## 3. Results

### Growth Performance

The sunflower meal inclusion rate, effect of exogenous enzymes and interaction between sunflower levels on growth performance of broiler chicks are presented in Table 5 and Table 6. Mortality recorded in this study was low (<3%) and unrelated to dietary treatments. During the pre-starter period (1 to 9 d), SFM inclusion had no effect in any of the studied parameters. BWG, FI and FCR did not significant differ due to the different inclusion levels of SFM. Enzyme supplementation had no effect in all studied growth performance parameters in the pre-starter phase.

During the grower phase, there was no significant differences in BWG, FI and FCR due to diet type. The same trend was observed for enzyme addition. However, there was a tendency for enzyme addition on BWG (*p* = 0.050) during this period; with XAP addition resulting to the highest BWG (0.983 kg), whereas the addition of XBP resulted in the lowest BWG (0.949 kg), even though not significantly different from each other. There was also no diet type interaction by enzyme for all studied performance parameters in the grower phase.

In the finisher phase, diet type, enzyme and diet and enzyme interaction only influenced BWG and not FI and FCR. BSH recorded a higher BWG (1.84 kg vs. 1.82 kg). XAP and X enzymes resulted in significantly (*p* < 0.05) higher body weight gains than the rest of the enzyme treatments.

In the post-finisher phase, diet type influenced all the studied parameters except for FCR. BSH had significantly (*p* < 0.05) higher BWG (2.64 kg vs. 2.62 kg) and FI (3.71 kg vs. 3.67 kg) than BSL in this phase. Enzyme addition had an effect in all studied parameters during the post-finisher phase, with xylanase addition resulting in significantly higher BWG (2.66 kg), FI (3.74 kg) and FCR (1.40). BSH diets supplemented with XAP recorded the highest body weight gain (2.69 kg), whereas BSL diets supplemented with XBP recorded the least BWG (2.58 kg).

## 4. Discussion

The current study investigated de-hulled sunflower meal inclusion rate and the effect of exogenous enzymes on growth performance of broiler chickens grown to 35 d. Previous studies show that responses of broiler chicks to SFM depend on its chemical composition, as well as the type of diet and enzymes supplemented. In the current study, during the pre-starter phase, increasing SFM inclusion did not affect any of the studied parameters. Our results are partially in agreement with those of [6], who only observed significance difference for the FCR, but not for BWG and FI in their study. Likewise, Ref. [22] did not find any significance difference in FI as influenced by SFM or enzyme addition, whereas enzyme supplementation influenced FCR and BWG in the grower phase. This differs to our study because during the grower phase, no significant differences were observed amongst all studied parameters; even FCR was not influenced by enzyme supplementation. The results on FCR may be an indication of the implication of non-starch polysaccharides on FCR [26]. Lower metabolizable energy tend to reduce growth performance of broiler chickens due to high concentrations of NSPs, which has an inverse effect on dietary energy levels. To counter that effect, in this study, metabolizable energy was added to some of the dietary treatments to see if there would be any variations in response. Treatments diets with additional energy performed better, placing our findings in sync with those reported by [35] who improved the nutritional value of high SFM meal for laying hens by adding oil and correcting for energy deficiencies to improve growth performance.

XAP enzyme supplementation to de-hulled SFM overall improved growth performance. We speculate that the observed increase in BWG, FI and FCR of the birds fed SFM supplemented with XAP may have been observed as a result of enhancement of other physiological and metabolic processes [30]. In addition to increasing nutrient digestibility, carbohydrases have been suggested to depolymerize complex NSPs, hence releasing fermentable xylo-, galacto-, manno-, or gluco-oligomers that have prebiotic effects from health promoting bacterial (*Lactobacillus*, *Bifidobacterium*) proliferation [30,36]. The consequence would be further increases in energy concentration and the enhancement of nutrient utilization and absorption [30,36].

Similarly, Ref. [16] observed an improvement in BWG and FCR in the finisher phase due to enzyme supplementation. Our study differs in terms of the results on FI, in that we also observed a significance difference on the diets because of SFM type and enzyme supplementation. Interestingly, Ref. [16] used a multi-enzyme consisting of xylanase, protease and amylase, a combination that we also adopted in the current study. It is therefore conceivable that the addition of XAP and X in our study resulted in higher BWG, FI and FCR’s in the post-finisher phase, like what was recorded to the overall study by the same authors, except for FI, which was not significantly affected in their study.

Additionally, an improvement in BWG and FCR was recorded elsewhere due to the addition of enzyme blend (cellulase, β-glucanase and xylanase) in diets containing 6% and 8% SFM in the grower and 10% SFM in the finisher, where enzyme supplementation started from the grower phase to the finisher phase [22]. In our study, enzyme supplementation resumed at the pre-starter phase. Body weight gain was only significantly (*p* < 0.05) improved when de-hulled sunflower meal was included in the diet at 13%, with the supplementation of enzyme.

In our study, the inclusion of dehulled-SFM at a higher levels (13–13.5%) resulted in higher BWG (13% and 13.5%), and FI (13.5%) due to enzyme supplementation. According to [37], higher levels of insoluble fibre in poultry diets has always been associated with negative effects of reduction of nutrient digestibility, absorption and feed intake, thus feeding diets rich in fibre in poultry tend to increase feed intake as a way of compensation for the reduced concentration of nutrients, mainly energy level, in the diets. Enzyme addition in the de-hulled SFM based diet was intended for the degradation of NSP and thereby facilitate the nutrient absorption and improve weight gain. These appeared to be true in our study because there was an improvement in BWG due to enzyme supplementation. The improvement could be a result of exogenous enzymes eliminating adverse and/or deleterious effects or impacts of anti-nutritional compounds, thus enhancing the nutrients utilization by the birds, particularly energy and amino acids which in turn translated into better broiler production performance observed in our study [16].

Chickens are generally unable to breakdown phytate and NSP that is present in most raw materials of plant origin due to their inability to secrete phytase and NSP-hydrolyzing enzymes [38]. Thus, they need exogenous enzymes to act upon the fibrous material more effectively by breaking the polymeric chain, thereby improving their nutritive value and reducing gut viscosity. In view of this, our study incorporated phytase enzyme with additional NSP-hydrolyzing enzymes (X, XB, XBP and XAP) to see their efficacy in breaking down the polymeric chains found in de-hulled SFM based diets. The activity was only determined by growth performance in our study, hence based on these, the results suggest that the effect of enzymes was not similar, only xylanase and XAP showed beneficial effect over the others overall. Xylanase showed significant improvements (*p* < 0.05) for all studied parameters in the post finisher phase. Enzyme effects differ according diet composition (target substrate), the age of the chickens and the dose of the enzymes [39]. Our study did not analyze the fractions of the substrates; hence, we could not ascertain the specific enzymes to target predominant substrates. According to [40], a consortium or a combination of enzymes offers greater benefits than when a single enzyme is used due to their synergistic effect on the various substrates. Our choice of enzymes was informed much by literature about the common substrates found in SFM (arabinoxylans, pectins, beta-glucans etc) [11,40]. Improved BWG and FCR were observed in birds grown of 1–21 d suggesting that more benefits are achieved at the early starter phase [39]. However, these studies administered their enzymes in drinking water whereas enzymes in the current study enzymes were administered in the feed and we observed benefits at the post finisher phase.

Successful use of enzymes in broiler diets is restricted by the cost-benefit ratio [39]. However, such analysis was not the basis for our study. Actually, reducing the cost of feed offers the reserve for cost reduction, and this is achieved by reducing the cost of the protein component of the diet [13]. By increasing the levels of dehulled SFM (13–13.5%) in the diet, SBM inclusion was partially replaced as the most expensive traditional protein source. Therefore, we inherently realized the economics of using enzymes as evidenced through overall improvements in production performance of our study. Factoring the prices of enzymes, we could still realize the beneficial effects since using SFM in the diet implies that it must be supplemented with synthetic oil and lysine, thus making the feed not cost effective [13].

Nevertheless, there are many cases in which enzyme supplementation to SFM containing diets did not improve of all studied growth parameters [6,27,28,29]. In some instances, exogenous enzymes use resulted in worse performance parameters [26], whereas in some studies, including ours, it resulted in improved growth performance parameters [16,22,23,24,30]. Such discrepancies in the results recorded in literature are multifaceted, and may be attributed to the quality of SFM processing, variety of experimental birds, experimental periods (21, 35 or 42 d), and diet composition [30]. In our study we used Ross 308 males whereas some of the studies on literature used Hubbard [16], Cobb 500 [3], Arbor Acres [39], layers [41] and in some instances, quails [29]. Our experimental period was 35 d and enzyme supplementation began at day-old with birds on iso-caloric and iso-nitrogenous diets.

According to [42], exogenous enzymes helps in the disruption of cell wall integrity in plant based ingredients, and the disruption of the cage effect, results in the subsequent release of nutrients that were previously housed by the cell, thus making them available to the animal for use. Additionally, enzymes use has been shown to be responsible for the breaking down of NSP’s, improvement of nutrient digestibility due to a reduction of the gut viscosity and thereby improving the growth performance and uniformity of birds flock [25].

The non-starch polysaccharides consists of a portion that is water-soluble that is responsible for the formation of a viscous texture in the gastro-intestinal tract (GIT), hence decreasing gut performance of the bird [1]. β-glucans mostly possess a negative effect on nutrients, especially protein and starch utilization, leading to highly viscous condition in the small intestines which are associated with nutrient absorption reduction due to negative changes in the GIT microflora [1,43]. According to [44], cereal β-glucans are not digested by the monogastric animal’s endogenous enzymes and have a negative effect on bird performance and health. There was no significant differences amongst the PC, NC, XB and XBP treatments in terms of overall BWG. However, numerically, our results show that supplementation with XBP enzyme led to poor growth performance, suggesting that the cereal β-glucans were not hydrolyzed efficiently by the enzyme combination. Probably these may be because enzyme XBP did not promote improvement in overall epithelial cell arrangement and villus height, thereby limiting nutrient absorption [43]. Worth mentioning is that we did not do intestinal morphology in our study, so we are making assumptions on the possibilities of such occurrence.

## 5. Conclusions

The results suggest that SFM can be increased to at least 13% and 13.5% finisher and post-finisher broiler chickens’ diets without negatively affecting performance, and that the addition of xylanase, amylase and protease enzyme cocktail and xylanase enzymes can improve body weight gain and feed intake of broilers grown to 35 d of age. This will result in a substantial decrease in feed price while yielding similar results. The beneficial effect of the enzyme cocktail containing xylanase, amylase and protease could be associated with the hydrolysis of the different constituents of NSP’s found in de-hulled SFM-based diets, thus achieving the study’s objectives of increasing de-hulled SFM inclusion levels without impairing body performances in broiler chickens.

## Figures and Tables

**Table 1:** Composition and calculated nutrients (%) of the basal diets for the pre-starter (1–9 d) and grower phase (10–20 d).

Ingredients as Fed	Pre-Starter				Grower			
	BSL+	BSL−	BSH+	BSH−	BSL+	BSL−	BSH+	BSH−
Maize	56.9	55.1	54.9	53.0	64.5	62.7	61.3	59.4
Soya oilcake	30.1	30.4	26.8	27.1	23.8	24.1	18.7	19
Full fat soya	4.7	4.7	4.7	4.7	5	5	5	5
De-hulled sunflower oilcake (38%)	3	3	7.5	7.5	3	3	10	10
Gluten 60	-	-	-	-	-	-	-	-
Synthetic Lysine (78%)	0.29	0.29	0.35	0.34	0.31	0.30	0.39	0.38
Valine	0.01	0.01	0.01	0.01	0.02	0.02	0.02	0.02
DL-Methionine (78%)	0.30	0.30	0.30	0.30	0.29	0.29	0.27	0.27
Threonine (98%)	0.15	0.15	0.15	0.15	0.07	0.07	0.07	0.07
Oil crude soya (Degummed)	0.50	2.05	1.35	2.90	0.59	2.14	1.93	3.48
Feed lime	1.79	1.79	1.77	1.77	1.13	1.12	1.10	1.10
Salt	0.16	0.16	0.13	0.14	0.15	0.15	0.11	0.12
Monocalcium phosphate	1.35	1.35	1.34	1.34	0.58	0.58	0.56	0.56
Phytase 1000	0.015	0.015	0.015	0.015	0.015	0.015	0.015	0.015

BSL+: low de-hulled sunflower meal + 80 kcal; BSL−: low de-hulled sunflower meal − 80 kcal; BSH+: high de-hulled sunflower meal + 80 kcal; BSH−: high de-hulled sunflower meal − 80 kcal.

**Table 2:** Composition and calculated nutrients (%) of the basal diets for the finisher (20–28 d) and post-finisher phase (29–35 d).

Ingredients as Fed	Finisher				Post-Finisher			
	BSL+	BSL−	BSH+	BSH−	BSL+	BSL−	BSH+	BSH−
Maize	63.3	66.1	59.1	62.1	65.5	68.3	60.7	63.9
Soya oilcake	26.6	25.8	12	11	24.9	24.1	8.73	7.37
Full fat soya	2	1.9	12	11	1.5	1.46	12	12
De-hulled Sunflower oilcake (38%)	3	3	13	13	3	3	13.5	13.5
Gluten 60	0	0	0.6	0.6	0	0	0	0
Synthetic Lysine (78%)	0.30	0.30	0.42	0.43	0.29	0.29	0.41	0.42
Valine	0.03	0.02	0.03	0.02	0.02	0.01	0.02	0.02
DL-Methionine (78%)	0.27	0.27	0.25	0.24	0.26	0.24	0.23	0.22
Threonine (98%)	0.07	0.06	0.08	0.07	0.06	0.05	0.08	0.07
Oil crude soya (Degummed)	2.31	0.5	2.19	0.5	2.32	0.5	2.42	0.5
Feed lime	1.07	1.07	1.00	1.01	1.07	1.07	1.00	1.00
Salt	0.15	0.15	0.10	0.09	0.15	0.15	0.10	0.10
Monocalcium phosphate	0.34	0.35	0.34	0.34	0.37	0.37	0.35	0.36
Phytase 1000	0.015	0.015	0.015	0.015	0.015	0.015	0.015	0.015

BSL+: low de-hulled sunflower meal + 80 kcal; BSL−: low de-hulled sunflower meal − 80 kcal; BSH+: high de-hulled sunflower meal + 80 kcal, BSH−: high de-hulled sunflower meal − 80 kcal.

**Table 3:** Calculated and analysed basal dietary treatments formulations used in the experiment (pre-starter and grower).

Ingredients (%)	Pre-Starter				Grower			
	BSL+	BSL−	BSH+	BSH−	BSL+	BSL−	BSH+	BSH−
Calculated nutrients (DM%)								
Moisture	10.40	10.22	10.22	10.04	10.67	10.40	10.22	11.53
ME Poultry (MJ/kg)	12.01	12.37	12.02	12.38	12.54	12.89	12.55	12.90
Crude protein	21.91	21.90	21.95	21.94	19.65	19.71	19.34	19.54
Fat	4.14	5.62	4.87	6.35	4.40	5.89	5.55	7.03
Crude fibre	3.01	2.98	3.89	3.87	2.95	2.93	4.33	4.30
Calcium	1.09	1.09	1.09	1.09	0.72	0.72	0.72	0.72
Digestible Phosphorus	0.52	0.52	0.52	0.52	0.38	0.38	0.38	0.38
Analysed nutrients (DM%)								
Dry matter	90.92	90.42	91.58	91.40	91.15	91.23	90.93	89.61
Moisture	9.08	9.58	8.42	8.60	8.85	8.77	9.07	10.39
Crude fibre	2.97	3.02	3.92	3.85	2.90	2.88	4.36	4.37
Ether extract	4.12	5.55	4.79	6.31	4.46	5.93	5.52	6.66
Ash	5.98	5.89	6.34	6.10	4.75	4.46	4.33	4.49
Crude protein	23.03	23.18	23.56	23.18	19.57	19.57	19.28	19.18

BSL+: low de-hulled sunflower meal + 80 kcal; BSL−: low de-hulled sunflower meal − 80 kcal; BSH+: high de-hulled sunflower meal + 80 kcal; BSH−: high de-hulled sunflower meal − 80 kcal; ME, metabolizable energy.

**Table 4:** Calculated and analysed basal dietary treatments formulations used in the experiment (finisher and post-finisher).

Ingredients (%)	Finisher				Post-Finisher			
	BSL+	BSL−	BSH+	BSH−	BSL+	BSL−	BSH+	BSH−
Calculated nutrients (DM%)								
Moisture	11.53	11.78	10.65	10.94	11.62	11.86	10.69	10.95
ME Poultry (MJ/kg)	12.68	12.32	12.77	12.41	12.75	12.72	12.85	12.81
Crude protein	19.34	19.54	19.60	19.71	18.57	18.76	18.77	18.84
Fat	5.57	3.83	6.91	5.14	5.52	3.78	7.16	5.33
Crude fibre	2.90	2.92	5.00	5.00	2.86	2.89	5.09	5.11
Calcium	0.65	0.65	0.65	0.65	0.65	0.65	0.65	0.65
Digestible phosphorus	0.34	0.34	0.34	0.34	0.34	0.34	0.34	0.34
Analysed nutrients (DM%)								
Dry matter	91.18	89.96	91.44	90.65	90.97	90.54	91.62	90.25
Moisture	8.82	10.04	8.56	90.35	9.03	9.46	8.38	9.75
Crude fibre	2.93	2.89	5.02	5.05	2.89	3.02	5.01	5.57
Ether extract	5.50	3.91	6.88	5.16	5.55	3.83	7.15	5.36
Ash	4.22	4.17	4.12	4.05	4.1	3.99	4.13	4.03
Crude protein	19.61	19.42	19.57	19.23	18.55	18.41	18.37	18.04

BSL+: low de-hulled sunflower meal + 80 kcal; BSL−: low de-hulled sunflower meal − 80 kcal; BSH+: high de-hulled sunflower meal + 80 kcal; BSH−: high de-hulled sunflower meal − 80 kcal; ME, metabolizable energy.

**Table 5:** Cumulative growth performance for broiler’s fed SFM-based diets with different exogenous enzymes from pre-starter and grower phase; BW (kg), FI (kg), FCR (kg/kg).

^1^ Diet Type	Enzyme	Pre-Starter Phase (0–9 d)			Grower Phase (0–20 d)		
		BWG	FI	FCR	BWG	FI	FCR
BSL	NC	0.22	0.25	1.14	0.96	1.06	1.10
	PC	0.21	0.24	1.16	0.94	1.03	1.11
	X	0.21	0.25	1.16	0.96	1.08	1.13
	XAP	0.22	0.25	1.14	0.97	1.09	1.13
	XB	0.22	0.25	1.16	0.96	1.07	1.13
	XBP	0.21	0.21	1.15	0.94	1.05	1.12
BSH	NC	0.22	0.25	1.17	0.97	1.09	1.13
	PC	0.21	0.26	1.19	0.97	1.11	1.14
	X	0.21	0.25	1.19	0.96	1.08	1.13
	XAP	0.22	0.25	1.16	0.10	1.10	1.11
	XB	0.22	0.26	1.17	0.96	1.09	1.13
	XBP	0.22	0.25	1.16	0.95	1.08	1.13
^2^ SEM		0.015	0.025	0.015	0.017	0.027	0.017
Main effects							
Diet type							
BSL		0.22	0.25	1.15	0.95	1.07	1.12
BSH		0.22	0.25	1.17	0.97	1.09	1.13
Enzyme							
NC		0.22	0.25	1.15	0.97	1.08	1.12
PC		0.21	0.25	1.18	0.95	1.07	1.13
X		0.21	0.25	1.17	0.96	1.08	1.13
XAP		0.22	0.25	1.15	0.98	1.10	1.12
XB		0.22	0.25	1.17	0.96	1.08	1.13
XBP		0.21	0.24	1.15	0.95	1.07	1.12
*p*-values							
Diet type		0.951	0.722	0.052	0.160	0.129	0.428
Enzyme		0.562	0.733	0.123	0.050	0.215	0.417
Diet × Enzyme		0.615	0.663	0.274	0.120	0.062	0.083

^1^ Diets: BSL: low sunflower meal; BSH: high sunflower meal; NC: negative control (basal diet − 80 kcal AMEn); PC: positive control (basal diet + 80 kcal AMEn); X: Control diet in which xylanase enzyme was added; XAP: control diet + xylanase + amylase + protease enzyme combination; XB: control diet with xylanase + beta-glucanase; XBP: control diets with xylanase + beta-glucanase + protease enzymes. BWG: Body weight gain; FI: Feed intake; FCR: Feed conversion ratio. ^2^ SEM, Standard error mean.

**Table 6:** Cumulative growth performance for broiler’s fed SFM-based diets with different exogenous enzymes from grower phase and overall, 35-day period BW (kg), FI (kg), FCR (kg/kg).

^1^ Diet Type	Enzyme	Finisher Phase (0–28 d)			Post-Finisher Phase (0–35 d)		
		BWG	FI	FCR	BWG	FI	FCR
BSL	NC	1.80 ^b^	2.25 ^bc^	1.25	2.59 ^cd^	3.64 ^b^	1.42
	PC	1.79 ^b^	2.20 ^c^	1.23	2.56 ^d^	3.58 ^b^	1.42
	X	1.83 ^ab^	2.29 ^ab^	1.25	2.66 ^ab^	3.75 ^a^	1.40
	XAP	1.86 ^a^	2.30 ^ab^	1.24	2.69 ^a^	3.73 ^a^	1.37
	XB	1.83 ^ab^	2.28 ^ab^	1.25	2.63 ^bc^	3.65 ^b^	1.39
	XBP	1.82 ^ab^	2.22 ^bc^	1.22	2.61 ^bc^	3.64 ^b^	1.40
BSH	NC	1.84	2.30	1.25	2.65 ^ab^	3.74 ^a^	1.41 ^a^
	PC	1.86	2.29	1.23	2.67 ^a^	3.66 ^bc^	1.36 ^b^
	X	1.84	2.27	1.23	2.66 ^ab^	3.73 ^ab^	1.40 ^ab^
	XAP	1.86	2.29	1.23	2.69 ^a^	3.74 ^a^	1.37 ^ab^
	XB	1.83	2.30	1.26	2.62 ^bc^	3.73 ^ab^	1.43 ^a^
	XBP	1.82	2.27	1.25	2.58 ^c^	3.64 ^c^	1.42 ^a^
^2^ SEM		0.018	0.029	0.018	0.018	0.025	0.018
Main effects							
Diet type							
BSL		1.82 ^b^	2.26	1.24	2.62 ^b^	3.67 ^b^	1.40
BSH		1.84 ^a^	2.29	1.24	2.64 ^a^	3.71 ^a^	1.40
Enzyme							
NC		1.82 ^b^	2.27	1.25	2.62 ^b^	3.69 ^a^	1.41 ^a^
PC		1.82 ^b^	2.25	1.23	2.61 ^b^	3.62 ^b^	1.39 ^b^
X		1.84 ^ab^	2.28	1.24	2.66 ^a^	3.74 ^a^	1.40 ^a^
XAP		1.86 ^a^	2.29	1.23	2.69 ^a^	3.74 ^a^	1.37 ^bc^
XB		1.83 ^ab^	2.29	1.25	2.62 ^b^	3.69 ^a^	1.41 ^a^
XBP		1.82 ^b^	2.25	1.24	2.60 ^b^	3.64 ^b^	1.41 ^a^
*p*-values							
Diet type		0.042	0.07	0.866	0.028	0.01	0.796
Enzyme		0.031	0.096	0.219	0.000	0.001	0.017
Diet × Enzyme		0.003	0.001	0.113	0.000	0.001	0.011

^1^ Diets: BSL: low sunflower meal; BSH: high sunflower meal; NC: negative control (basal diet − 80 kcal AMEn); PC: positive control (basal diet + 80 kcal AMEn); X: Control diet in which xylanase enzyme was added; XAP: control diet + xylanase + amylase + protease enzyme combination; XB: control diet with xylanase + beta-glucanase; XBP: control diets with xylanase + beta-glucanase + protease enzymes. BWG: Body weight gain; FI: Feed intake; FCR: Feed conversion ratio. ^2^ SEM, Standard error mean; ^a,b,c,d^ In a row, dietary treatment means with common superscripts do not differ (*p* < 0.05).
